# Oral health evaluation in special needs individuals

**DOI:** 10.1590/S1679-45082016AO3712

**Published:** 2016

**Authors:** Danielle de Moraes Pini, Paula Cristina Gil Ritter Fröhlich, Lilian Rigo

**Affiliations:** 1 Faculdade Meridional, Passo Fundo, RS, Brazil.

**Keywords:** Mouth diseases, Disabled persons, Oral hygiene

## Abstract

**Objective:**

To identify the prevalence of the main oral problems present in special needs children and to relate the underlying conditions with the clinical and demographic variables.

**Methods:**

The study was based on the physical examination of 47 students from the *Associação de Pais e Amigos dos Excepcionais* diagnosed as Down syndrome, cerebral palsy and intellectual *deficit*. For data collection, we used a self-administered questionnaire that included indices of dental caries and oral hygiene, Angle classification, malposition of dental groups and oral hygiene habits.

**Results:**

The predominant age group was 12-25 years (46.8%) and most patients were male (55.3%). Regarding daily brushing, 63.8% reported brushing their teeth three times a day, and 85.1% did it by themselves. A total of 48.9% were rated as Angle class I, and 25.5% had no type of malocclusion. A high dental carries index (decayed, missing, filled >10) was observed in 44.7%, and 53.2% had inadequate oral hygiene (zero to 1.16). There was a statistically significant difference between cerebral palsy and the act of the participants brushing their teeth by themselves.

**Conclusion:**

There was a high decayed-missing-filled teeth index and malocclusion class I, as well as inadequate oral hygiene. The type of underlying condition of the participants influenced the act of brushing teeth by themselves.

## RESUMO

**Objetivo:**

Conhecer a prevalência dos principais problemas bucais em crianças com necessidades especiais, e relacionar as doenças de base com variáveis clínicas e demográficas.

**Métodos:**

O estudo foi realizado a partir de exame clínico em 47 alunos da Associação de Pais e Amigos dos Excepcionais com diagnóstico médico de síndrome de Down, paralisia cerebral e *deficit* intelectual. Para a coleta de dados, foi utilizado um questionário autoaplicativo com índices de cárie dentária e higiene oral, classificação de Angle, malposição de grupos dentários e hábitos de higiene oral.

**Resultados:**

A faixa etária predominante foi de 12 a 25 anos (46,8%) e a maioria era do sexo masculino (55,3%). Em relação à escovação dentária, 63,8% relataram escovar os dentes três vezes ao dia, sendo que 85,1% realizavam-na sozinhos. Constatou-se que 48,9% dos examinados apresentavam uma classificação de Angle tipo I e 25,5% não apresentavam qualquer tipo de maloclusão. Os avaliados (44,7%) apresentaram alto índice de cárie dentária (cariados, perdidos e obturados >10) e 53,2% apresentaram higiene oral inadequada (zero a 1,16). Houve diferença estatisticamente significativa entre a paralisia cerebral e o ato de escovar os dentes sozinho.

**Conclusão:**

Constataram-se altos índices de cárie e de maloclusão classe I, além de inadequada higiene oral. Houve influência do tipo de patologia de base na realização do ato de escovar os dentes sozinhos.

## INTRODUCTION

According to data from the United Nations, temporary or permanent disabilities affect 10% of the population in developing countries. Thus we can say that in Brazil, which has a population of 205,129 million people, approximately 14,700 million of them have some sort of disability, distributed as mental (50%), physical (20%), auditory (15%), multiple (10%), and visual (5%).^(1)^ The oral condition of special needs patients may be directly and indirectly related to their physical or mental disorders.

The concept of “special needs patients” is any individual, adult or child, whose physical, intellectual, social, or emotional skills fall outside of what is considered normal regarding growth and development standards; thus they cannot receive standard education and require special and supplementary instruction throughout their lives.^(2)^

A mental disability is a state of functional limitation that is below the general average in any of the areas of human functionality and most importantly in the adaptation to their surroundings. According to the fourth edition of the Diagnostic and Statistical Manual of Mental Disorders-IV (DSM-IV) and the American Association on Mental Retardation, a below average intellectual ability affects at least two of the following areas: communication, self-care, house chores, social skills, interpersonal relationships, use of community resources, self-sufficiency, academic skills, work, entertainment, health and safety, and leisure administration.^(3)^

Cerebral palsy (CP) is a permanent condition, a non-progressive stable lesion that starts in the pre-, peri- or postnatal periods, and results in poor development of motor skills and intellectual disability of multifactorial etiology.^(3)^ Cerebral palsy comprises a group of permanent development, posture and movement disorders, which limit activities attributed to non-progressive conditions that occur during fetal development or in the child brain. Motor disorders are frequently accompanied by sensorial, cognitive, perceptive, communicative and behavioral alterations, as well as epilepsy and secondary musculoskeletal problems.^(4)^

Down syndrome (DS), or trisomy of chromosome 21, is a multisystemic congenital disease, first described by Langdon Down, in 1866. It is the most common congenital mental anomaly and includes several mental and behavioral alterations and physical malformations, including oral ones.^(5)^

Dental problems are common in these patients, with dental caries and gingivitis as the biggest concerns. These patients’ difficulty to keep adequate oral hygiene is enough to explain the high incidence of these problems, and other issues may also be present, such as mouth breathing, occlusion abnormality, cariogenic diet, and side-effects of medications.^(6)^

The participation of a dental professional is extremely important in the rehabilitation and integration of these patients in the social environment. This professional must be knowledgeable not only in their field, but must also in multidisciplinary areas. This assistance must be encouraged so that the care given to these patients happens in an integrated way (Physical Therapy, Psychology, Speech Therapy, Neurology, Dentistry, Nursing, Occupational Therapy, among others) with the patients’ well-being as the ultimate goal.

This research is relevant because, from its results, we can propose oral hygiene promotion and prevention programs for special needs children. The importance of the work also comes from the fact that there is very little research of this kind.

## OBJECTIVE

To understand the prevalence of the main oral problems in special needs children and the relation between underlying conditions and clinical and demographic variables.

## METHODS

### Sample and study design

A quantitative cross-sectional study, with a non-probability sample composed by 47 students aged 12 to 60 years, of both sexes, and obtained by convenience. A consent form was sent to all 61 guardians of the students from the *Associação de Pais e Amigos dos Excepcionais* (APAE). Of the 61 forms, 8 were returned to us illegible, and the students’ name could not be identified, and 6 students did not want to participate on the day of the clinical exam; therefore, we had a final sample of 47 students.

The 47 students attended APAE in the city of Passo Fundo, in the State of Rio Grande do Sul, and had the following conditions: DS, intellectual *deficit*, and CP. Passo Fundo is the largest city in the north region of the State. According to data from the Brazilian *Instituto Brasileiro de Geografia e Estatística* (IBGE) from 2014, the population of the city was estimated at 200 thousand people.^(7)^ Established in 1967, APAE is a civil, philanthropic, non-profit organization for care, cultural, health and education purposes. It assists people with intellectual and/or multiple disabilities, seeking their inclusion in the job market through several activities performed in the organization.^(8)^

Before the intraoral clinical exams, the researcher followed the norms of the World Health Organization (WHO), examining 15% of the sample (seven students) twice in consecutive days, so that the interexaminer calibration could be verified through Kappa test, whose measure of agreement was 0.89.

### Data collection instruments

The data was collected through a clinical exam in 47 students of both sexes, aged between 12 and 60 years, in March of 2015, using indices established by the WHO.^(9)^ The guardians were given free consent forms so that students could participate in the research. The research was approved by the organization through an authorization form. Thus, the work was part of the project approved by the Research Ethics Committee under protocol number, CAAE: 0033.0.362.000-09, opinion 0033/09.

To facilitate data collection, we used a self-administered questionnaire with items to be analysed for each student. The students were classified according to the manual from WHO. The exams were performed in the dental office of the APAE facilities. For the clinical exam, we used gloves, masks, goggles, mouth mirrors, and exploratory probes. Periodontal condition was assessed using the indicator Simplified Oral Hygiene Index (OHI-S) proposed by Greene et al.,^(10)^ which measures the existence of plaque and calculates the vestibular surface of 11, 31, 16, and 26 (upper right central incisor, left lower central incisor, upper first molars) and the lingual surface of 36 and 46 (first lower molars).

In the absence of the teeth required for the exam, or if teeth had carries or fillings, they were substituted by the subsequent tooth. The first molars were substituted for the second or third molars, and central incisors were replaced by the same teeth on the opposite side. Plaque and calculus indices were calculated separately though the sum of degrees attributed, whose result was divided by the number of examined surfaces.

The results were classified according to the obtained values, being from 0 to 1 for satisfactory oral hygiene; from 1.1 to 2 for regular oral hygiene; from 2.1 to 3 for deficient oral hygiene; and ≥3.1 for terrible oral hygiene.

Regarding dental cavities, we used the decayed-missing-filled teeth (DMFT) index, proposed by Klein et al.^(11)^ To obtain the DMFT index, we used an exploratory probe and a flat mouth mirror, under artificial light, after brushing. The DMFT index measures the attack of caries on permanent dentition. Its initials represent teeth that are, respectively, decayed (D), missing (M), filled (F), and the unit of measurement, which is teeth (T). The teeth that are “missing” are subdivided into extracted (E) and recommended extraction (rE).

According to the index values, they were classified into: very low DMFT (0.0 to 1.1), low DMFT (1.2 to 2.6), moderate DMFT (2.7 to 4.4), high DMFT (4.5 to 6.5), and very high DMFT (6.6 or more).

Malocclusion was assessed by Angle classification,^(12)^ based on anteroposterior relations. Malocclusions were classified according to the first permanent molars. Class I, or neutroclusion, was used when the mesiobuccal cusp of the first upper molar occluded towards the mesiobuccal groove of the first lower molar. Class II, or distoclusion, was characterized by the distal position of the first lower molars in relation to the upper ones, in such a way that the mesiobuccal cusp of the first upper molar occluded mesially to the mesiobuccal groove of the first lower molar. Class III, or mesio-occlusion, was used when the first lower molar was mesially related to the upper one, in a way that the mesiobuccal cusp of the first upper molar distally occluded to the mesiobuccal groove of the first lower molar.

Regarding the malposition of tooth groups, we evaluated open anterior bite (when occluded, there is distancing of some teeth in the vertical dimension – in this bite, posterior teeth touch and anterior teeth are separated), anterior crossbite (when upper anterior teeth occlude to the lingual of lower teeth), posterior crossbite (abnormal relation, vestibular or lingual, of one or more maxillary teeth with one or more mandibular teeth – it may be unilateral or bilateral), overbite (decrease in the vertical dimension where the upper teeth cover more than 1/3 of lower teeth), and end-to-end bite (upper teeth do not cover the lower teeth – arches stay one above the other).

The questionnaire also contained questions on patients’ oral hygiene habits, how many times a day it was performed, and if they did it by themselves or had help.

### Statistical analysis of the data

Data were statistically analysed by the software Statistical Package for the Social Sciences (SPSS), version 20.0 for Windows. Statistical analysis followed a descriptive and inferential analysis of data. The test used allowed us to verify the relations between the variables, and the presentation was done in the way of frequencies by inferential analysis using the χ^2^ test. Significance level was set at 5% (p<0.05). As a dependent variable, we used the underlying conditions of the students examined (DS, CP, and intellectual *deficit*).

## RESULTS

Table 1 shows the results regarding the occurrence of all analysed variables. Of the 47 examined students, 36.2% had DS, 36.2% had CP, and the remaining 27.7% had intellectual *deficit*. The predominant age group was between 12 and 25 years, which represented 46.8% of sample. The majority were males (55.3%). Regarding daily brushing, 63.8% said to brush their teeth three times a day, and 85.1% said they brushed their teeth on their own. Of the patients examined, 48.9% were Angle class I, 46.8% were class II, and 25.5% did not present any type of malocclusion according to the malposition of dental groups. Among the evaluated patients, 53.2% had a DMFT index ≤10, and 53.2% presented an OHI-S index between zero and 1.16%.

**Table 1:** Demographic and clinical variables

Variables	n (%)
Condition	
Down syndrome	17 (36.2)
Intellectual deficit	13 (27.7)
Cerebral palsy	17 (36.2)
Sex	
Female	21 (44.7)
Male	26 (55.3)
Age group (years)	
12-25	22 (46.8)
26-40	17 (36.2)
41 or more	8 (17)
Daily brushing	
1	6 (12.8)
2	11 (23.4)
3 or more	30 (63.8)
Brushes teeth on their own	
Yes	40 (85.1)
No	7 (14.9)
Angle classification, class	
I	23 (48.9)
II	2 (4.3)
III	22 (46.8)
Malocclusion	
Anterior open bite	3 (6.4)
Anterior crossbite	8 (17)
End-to-end	10 (21.3)
Bilateral posterior crossbite	1 (2.1)
Left unilateral posterior crossbite	1 (2.1)
Right unilateral posterior crossbite	4 (8.5)
No malocclusion	12 (25.5)
Anterior crossbite and bilateral crossbite	5 (10.6)
Anterior crossbite and unilateral left crossbite	2 (4.3)
Anterior crossbite and right unilateral crossbite	1 (2.1)
DMFT category	
≤10	25 (53.2)
>10	21 (44.7)
OHI-S category	
0-1.16	25 (53.2)
1.33-3.0	22 (46.8)

DMFT: decayed-missing-filled teeth; OHI-S: simplified oral hygiene index.

**Figure 1:**
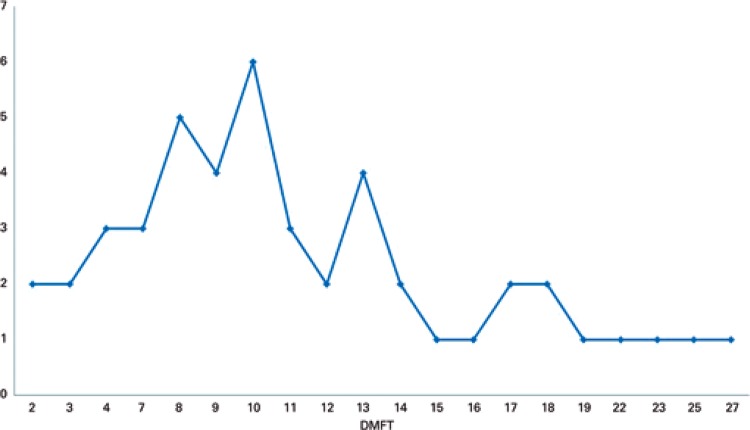
Mean of decayed, missing, filled teeth

**Figure 2:**
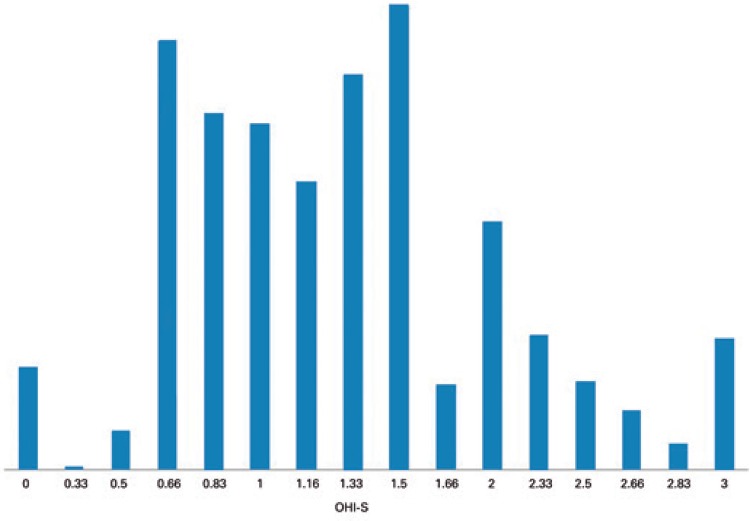
Simplified oral hygiene index

The data on DMFT, whose mean was 11 (standard deviation 5.2-16), are presented in figure 1. The data on OHI-S, whose mean was 1.28 (standard deviation 0.7-1.86), are presented in figure 2.

DMFT: decayed-missing-filled teeth.

OHI-S: Simplified Oral Hygiene Index (OHI-S).

Table 2 shows the bivariate analyses obtained through Fisher’s χ^2^ test used to test the equality and equivalence hypothesis between the proportions, in a confidence interval of 95%, with a significance level of 5%. There was a statistically significant difference between underlying conditions and the act of brushing teeth on their own (p=0.019), and 71.4% of individuals that did not brush on their own had CP as their underlying condition.

**Table 2:** Bivariate analysis between the independent variables and the underlying conditions

Independent variables	Underlying conditions				p value
	
	Down syndrome	Cerebral palsy	Intellectual *deficit*	Total	
	n (%)	n (%)	n (%)	n (%)	
Brush teeth on their own					
Yes	16 (94.11)	8 (61.53)	16 (94.11)	40 (100)	0.019*
No	1 (5.88)	5 (38.47)	1 (5.88)	7 (100)	
Total	17 (100)	13 (100)	17 (100)	47 (100)	
Sex					
Male	7 (41.17)	9 (69.23)	10 (58.82)	26 (100)	0.290
Female	10 (58.82)	4 (30.77)	7 (41.17)	21 (100)	
Total	17 (100)	13 (100)	17 (100)	47 (100)	
Age group, years					
12-25	9 (53.0)	7 (53.84)	6 (35.3)	22 (100)	0.516
26-40	6 (35.2)	5 (38.46)	6 (35.3)	17 (100)	
41-55	2 (11.7)	1 (7.69)	5 (29.40)	8 (100)	
Total	17 (100)	13 (100)	17 (100)	47 (100)	
Daily brushing					
1	2 (31.00)	3 (50.0)	1 (5.88)	6 (100)	0.726
2	4 (32.00)	3 (27.3)	4 (23.52)	11 (100)	
3	11 (36.7)	7 (23.3)	12 (70.58)	30 (100)	
Total	17 (100)	13 (100)	17 (100)	47 (100)	
DMFT category					
≤10	8 (47.05)	9 (69.23)	9 (52.95)	25 (100)	0.439
>10	9 (52.95)	4 (30.76)	8 (47.05)	22 (100)	
Total	17 (100)	13 (100)	17 (100)	47 (100)	
OHI-S category					
0-1.16	12 (70.58)	5 (38.46)	8 (47.05)	25 (100)	0.178
1.33-3.0	5 (29.42)	8 (61.53)	9 (52.95)	22 (100)	
Total	17 (100)	13 (100)	17 (100)	47 (100)	

*p<0.05: statistically significant difference. DMFT: decayed-missing-filled teet; OHI-S: Simplified Oral Hygiene Index.

## DISCUSSION

Besides systemic diseases and characteristics of certain conditions, we can consider oral diseases as one of the main problems affecting individuals with special needs, be it for their mental or motor condition.

Although the Brazilian government promotes a program called *Plano Nacional dos Direitos da Pessoa com Deficiência* (National Plan for the Rights of Persons with Disabilities), which includes the training of Primary Care professionals and qualification of the *Centro de Especialidades Odontológicas* (Center of Dental Specialties), it is still not enough to assist this population. There is a lack of advertising and accessibility to these facilities, lack of commitment and information of people responsible for these patients, and lack of trained professionals to perform patient care.^(13)^

According to Morales-Chávez et al.,^(14)^ every person requires appropriate dental assistance. In the case of special needs patients, the professionals must have broader knowledge, considering some deficiencies are associated to severe dental problems, such as bruxism, malocclusion, gingivitis and caries among others. Many of these diseases are often related to the patient’s diet or difficulty in performing adequate oral hygiene.

All students evaluated presented cavity indices considered very high, with a mean DMFT of 11. Down syndrome patients, unlike those with intellectual *deficit* and CP, presented oral characteristics associated with the syndrome. Regarding individuals with DS, Gonçalves et al.^(15)^ did a research in the DS Association of the city of Teresópolis, in the State of Rio de Janeiro, in which they assessed the DMFT index and periodontal health of 24 of their students with DS. The present study had different results from those obtained by other authors, since the students presented a low cavity index, with a mean of 5.27% in total, and a high periodontal disease index, and 59.25% of them presented some periodontal alteration. To decrease the high cavity index, it is necessary to implement health promotion strategies for these individuals and their caretakers to encourage better oral health.^(14)^

The present study evaluated the level of periodontal health through OHI-S, which was considered regular. A study conducted by Dávila et al.^(16)^ in the city of Morán, in Venezuela, with 60 students with DS and intellectual *deficit*, showed that a large part of them presented a high cavity index (75%), especially those with mild and moderate intellectual *deficit*. Regarding those with mild DS, 77.8% of those examined did not present cavities. Similarly to a study carried out by Lazzaretti et al.^(17)^ with 34 students enrolled in the *Centro de Apoio de Necessidades Especiais Paulo Schneider* (CANEPS) (Support Center for Special Needs Paulo Schneider), in the city of Barros Cassal, State of Rio Grande do Sul. These authors reported that, despite a sample that was insufficient for epidemiological conclusions, a very high cavity index was observed (88.2% of individuals), in a similar way to this study realized at APAE in the city of Passo Fundo, State of Rio Grande do Sul.

A study done by Vellappally et al.^(18)^ with children with several syndromes, including CP, DS and intellectual *deficit*, concluded that, of the 243 examined children, 93% presented some form of malocclusion requiring orthodontic treatment. The most common malocclusion was crowding of anterior teeth in 84.8%, followed by anterior mandibular irregularity ≥1mm, in 77.8%. In the present study, 25.5% of examined students did not present any kind of malocclusion.

Regarding Angle classification, 64.7% of DS patients were rated at class III, similarly to a study by Soares et al.,^(12)^ in the city of Teresina, State of Piauí, with only DS patients, in which 60% of students were also rated at Angle class III.

Regarding malocclusion, bilateral posterior crossbite was the most prevalent, affecting 52% of the sample. In the present study, other pathologies were evaluated, and end-to-end bite was the most prevalent, in 21.3% of all observed cases, followed by anterior crossbite, in 17%. Regarding DS patients in this study, bilateral posterior crossbite with anterior crossbite was the most prevalent in 29.4% of cases, followed by anterior crossbite, in 23.5% of cases. According to the data presented in both studies, we can confirm that there is a really higher prevalence of class III malocclusion for DS patients. Santos et al.^(19)^ stated the jaw is underdeveloped, with retrusion of the middle third of the face, thus explaining the predominance of class III.

Garcés et al.,^(20)^ reported inadequate oral hygiene as the major cause of periodontal disease in people with some sort of deficiency. The author reported that there is also a relation between the level of oral hygiene and the degree of deficiency. A study had a sample of 184 individuals with intellectual *deficit* enrolled in municipal schools in the city of Valdivia, in Chile.^(20)^ Among the individuals with mild intellectual *deficit*, 76.6% presented regular oral hygiene, and 7.1% presented good oral hygiene. However, of those with moderate intellectual *deficit*, 63.3% presented regular oral hygiene, and none had satisfactory oral hygiene. In the present study, OHI-S was regular among all patients examined, with a mean of 1.25.

In the present study, 63.8% of examined students reported they brush their teeth three times a day, and 85.1% said they do it on their own. The mean of daily brushing among female patients was 2.61 times per day, and among male patients, it was 2.42 times per day. In the study by Garcés et al.,^(20)^ only 3.7% of the patients examined had assistance to brush their teeth, and the average of brushing was 2.18 per day among the women – similarly to the present study – and 1.97 times per day for men.

As per Silva et al.,^(21)^ the salivary pH of DS patients was higher than of those without the syndrome and, as a consequence, they presented a higher buffer capacity, which leads to a lower incidence of caries. The incidence did not appear to be higher than in those without the syndrome. The results disagreed from those obtained by Fiorati et al.^(22)^ and Cogulu et al.,^(23)^ who demonstrated these individuals have a lower cavity index. This information was different from the results obtained in the present study, in which the cavity index assessed by DMFT was high, even in the city of Passo Fundo, where there is a program of fluoridation of drinking water established since 1975 (0.07ppm).

We must consider some factors that may affect the efficacy of treatments and preventive measures, such as lack of appropriate control, difficulty during dental assistance, underestimation of patient’s pain or treatment needs, communication problems and bad behavior. Garcés et al.^(20)^ demonstrated the need to establish a closer relationship with these patients to facilitate their care. We agree with the authors that not only caretakers but also the institutions should conduct oral hygiene programs to decrease the cavity and periodontal disease indices, considering these students spend many hours in the organizations, with no access to proper hygiene.

Regarding the bivariate analyses, the variable that presented a statistically significant difference was between the underlying condition and brushing teeth on their own: 71.4% of patients with CP were not able to brush teeth by themselves.

Students with CP who do not need help brushing their teeth did not present a difference in relation to the DMFT, as compared to those who did need assistance. This fact corroborates the necessity of teaching caretakers the correct way to perform the oral hygiene of these patients. As per Guerreiro et al.,^(24)^ the individuals with CP are given little dental attention due to the difficulty in handling them. The authors also mention that the low level of education and family income were factors that hindered access to and continuity of dental treatment.

As a limitation of the study, we can mention the lack of participation of students due to parents/caretakers who did not give consent. Because of this small participation, we suggest the use of a questionnaire directed at parents and/or caretakers, which could be a way to better understand dietary and family hygiene habits, as well as the degree of knowledge and importance given to oral health. According to the answers presented, we could develop lectures related to prevention and oral health promotion.

The patients, whether presenting mental or physical disabilities, can be difficult for dental surgeons to handle and treat. Due to the high number of patients who need special assistance, it is very important that dental surgeons know about the most frequent oral alterations and diseases to be able to offer adequate dental care to these individuals and be aware of their limitations.

From the results observed, we can suggest training brushers and professionals to teach about oral hygiene, especially after eating, as a method of prevention. It would be important to develop health promotion measures, such as lectures to instruct parents and caretakers, emphasizing the importance of good oral hygiene and the problems that can arise from its absence.

## CONCLUSION

We observed a high cavity index and inadequate oral hygiene among the participants. Moreover, according to Angle classification, there was a predominance of class I. The type of underlying condition had an influence on patients brushing their teeth by themselves, since most students do it without the assistance of parents or caretakers.
